# Interpersonal relationships and drug use over time among homeless people: a qualitative study

**DOI:** 10.1186/s12889-020-09880-2

**Published:** 2020-11-19

**Authors:** Marília Ignácio de Espíndola, André Bedendo, Eroy Aparecida da Silva, Ana Regina Noto

**Affiliations:** 1 NEPSIS - Research Center on Health and Substance Use, Sao Paulo, Brazil; 2 Department of Psychobiology, Universidade Federal de São Paulo – UNIFESP, Rua Botucatu, 862 – 1° Andar, Vila Clementino, SP 04023062 Sao Paulo, Brazil

**Keywords:** Homeless person, Interpersonal relationship, Social marginality, Social determinant of health, Substance-related disorders, Life cycle

## Abstract

**Background:**

Homelessness is one of the most severe forms of social exclusion and is an important public health issue. It is characterized by processes of weakening of interpersonal bonds. The objective of this study was, therefore, to elucidate how interpersonal relationships change over the life cycle of homeless drug and alcohol users.

**Method:**

We used a qualitative methodology. The participants were adults who had a history of homelessness and use of alcohol and other drugs. The interviews were semi structured and used a timeline instrument. All interview were audio recorded, transcribed, and submitted to thematic analysis.

**Results:**

Twenty individuals participated in the study. Reports on social exclusion over time stood out in respect of four main themes and their respective subthemes: Theme 1 – Childhood: instability upbringing, abuse, violence, and an absent or not very present father figure; Theme 2 – Adolescence: school dropout and failure; acceptance of gender and sexual orientation; birth of first child, living with a partner or getting married: Theme 3 – Adulthood: estrangement or conflicting relationship with family; health problems; drug trafficking and prostitution; Theme 4 – Cross-cutting factors: death of relatives and substance use.

**Conclusion:**

The results suggest that interpersonal relationships are permeated by successive breakups, conflicts and other events that start in childhood and can have a cumulative effect in later stages of life, and cross the subsequent phases. Substance abuse and dependence are mentioned as cross-cutting factors that intensify social exclusion in all stages of life.

**Supplementary Information:**

**Supplementary information** accompanies this paper at 10.1186/s12889-020-09880-2.

## Background

Even though housing is a basic human need [1–3], the number of homeless people (HP) around the world is significant. Data from the Organization for Economic Co-operation and Development (OECD) show that Canada, Germany and the United States have the largest homeless population when compared with other countries in the organization [4].

Brazilian data about homeless populations are scarce and incomplete [5, 6]. There has been only one national study about this population, and it was carried out in 2008 [7] with an estimate of homelessness being calculated using statistical models [5]. The total number of homeless people in Brazil was estimated to be 101,854 individuals [5]. However, with regard to the municipal level, São Paulo, unlike most cities, does have some recent figures. A census of 2015 reported that the homeless population of the city was 15,905 [8]. Among all the data on homelessness, there are some themes that stand out, including: low levels of education, the tendency for individuals living on the street not have strong family relationships but to live with friends or unrelated people, economic instability in relation to sources of income, and widespread and frequent use of psychoactive substances [8].

Defining homelessness is not a simple task, and most countries adopt a definition based on their own legislation [9]. However, in the last years, there has been an effort to develop a global definition [10], with a number of proposals to define homelessness by dividing it into three basic categories: 1) people without accommodation 2) people living in temporary accommodation or in crisis 3) people living in severely inadequate and/or insecure accommodation, with each of these categories having their respective subcategories [11]. The articulation of a global definition with different classifications for people living on the streets can help in the measurement of the problem and the development of public policies that address this population [9, 12].

The Brazilian definition is contained in legislative decree 7.053 [13] that created the National Policy for Social Inclusion of the Homeless Population, which defines this population segment as being heterogeneous, characterized by maximum poverty, fragility or interruption of family bonds, due to the lack of conventional regular housing and experiencing a process of social disaffiliation [13]. This concept is based on theoretical references of social exclusion and goes beyond the common concept, but it does not adopt a typology or classification for this population. The result of this is that the Brazilian government has little up-to-date official data on the actual number of homeless people in the country, further promoting the invisibility that these individuals suffer from in society [5].

Homelessness is considered a public health issue, since the precariousness of the public health system can be both a trigger for homelessness and an aggravating factor for the maintenance of the situation. It can also lead to new health problems or worsen pre-existing conditions [14–16]. Due to the prevalence of a number of infectious diseases and mental disorders identified in that population [17], we should also highlight the high mortality rate among HP, which is seven times higher than that in the general population [18]. The leading causes of death are infectious diseases, heart conditions and substance abuse, in addition to external factors such as accidents, suicide and homicide [17–20].

Among the various health problems associated with homelessness, mental disorders associated with substance abuse require special attention [19, 21–23], as they are closely related to the risk of becoming homeless [23–25]. Substance abuse or dependence, particularly regarding illicit drugs, is a predictor of homelessness among adults, and is also a risk factor for the chronicity of such conditions [26, 27].

Still in respect of health issues, we should stress that HP experience a process of social exclusion. This concept is considered one of the social determinants of health, being defined by the World Health Organization (WHO) as a multidimensional dynamic process related to the inequality in the relations of power, interacting in four dimensions: economic, cultural, political and social [28]. Social exclusion concerns the social relations and the barriers imposed by organizations which prevent individuals from having access to the means of survival and developing as citizens [29, 30].

Studies report that before an individual becomes homeless, ruptures occur, preceded by breakdowns in the interpersonal relations in the four dimensions we described previously [31]. Moreover, alcohol and other types of drug abuse or dependence are often considered to make this situation worse [32, 33]. In this context, it is necessary to highlight the ethnographic work by Auerswald and Eyre [34], in which the authors propose that substance use is the main obstacle to getting off the streets for homeless young people. However, there are no descriptions as to in which phases of the life cycle these situations happened. Auerswald and Eyre [34], and Carlson, Sugano [35] propose a model based on the life cycle of young people on the street. Our study presents a different proposal, one that extends from childhood to adulthood, focusing on interpersonal relationships. We can also highlight other recent studies that have touched on the topic with a quantitative approach, for example Brown, Goodman [36], and a qualitative approach, as in the study by Mabhala, Yohannes [37]; however, they did not focus on interpersonal relationships or describe the phases in which they occur. In addition, these studies focus on other populations, such as veterans [38], or older adults [6], and not specifically on homeless adults.

Therefore, this study aims to examine interpersonal relationships across the life cycle of the adult street population who are alcohol and other drug users, found in the urban areas of the city of São Paulo, Brazil.

## Methods

### Study design

The study has a qualitative exploratory design [39, 40]. The exploratory design is used when the object of study is little known and there are few studies in the accumulated scientific literature [41]. According to Patton [39], the qualitative exploratory method is used when there is a shortage of valid and acceptable measures. The use of this method is justified by the fact that the research question in this study is a pioneering one that aims to analyse and identify interpersonal relationships throughout the human development life cycle.

### Participants

The participants had to meet all three of the following inclusion criteria: being a homeless person in accordance with the definition contained in the Brazilian Decree 7.053 [13]; reporting current alcohol or other drugs use; and being over 18 years old (and, therefore, an adult according to Brazilian law). In this study, we did not include adolescents and children as we aimed to examine life events at different moments of the participants’ lives. In addition, underage individuals represent a minority of the homeless population and assessing this population would also impose additional ethical difficulties.

The locations chosen were three areas in the city of São Paulo (two in the central region and one in the south region). Individuals who were reported to have any cognitive disorder by a network of key informants (social workers, social educators, and psychologists) that would prevent their participation in the interview were excluded.

### Sampling and recruitment

The sampling strategy used was carried out with the support of a key informants. As described by Patton [39], key informants are professionals who have extensive experience with homeless people. These professionals were interviewed and indicated the people who met the inclusion and exclusion criteria for participating in the study. The interviewer went to the field with the key informants to find each participant who had been indicated by these professionals in order to invite them to participate in the study. It was explained that the entire interview would be recorded and that they would not be identified. Twenty people were interviewed, one did not accept the invitation and two interviews were excluded due to the low quality of the interviews. The Committee of Ethics in Research of the Universidade Federal de São Paulo approved the study (# 2.468.584).

### Data collection and setting

The data collection happened at three different times and in three distinct regions in city of São Paulo. The first was carried out in July 2018 in the central region of São Paulo. In this region, substance use in the open is frequent. The interviewer entered the area with the help of two key informants: a social worker, and a homeless person who was familiar with the area and its population.

The second took place in November 2018 in the Sé district, also in the central region of São Paulo. Four professional key informants from an NGO, a partner of the project, helped select the participants in this region. The interviews were carried out in the places where the homeless people who use alcohol and other drugs were approached, that is, on the street.

The third phase took place in April 2019, in a shelter in the southern region of São Paulo. The second author knew a professional who worked in the area and they helped to select the participants. The interviews took place in a room reserved for that purpose.

In the central region of the city, the fieldwork started in November 2017, with the data collection starting in July 2018. In one of the regions, the first author had an established relationship with six of the participants (*N* = 6). After first contact, all of the individuals were invited to take part in the research after receiving a brief explanation about the study. After agreeing to participate, they received an explanation of the objectives of the project and the recording started. Only one person refused to participate when informed that the interview would be audio recorded. The first author was responsible for conducting all the interviews. All interviews were conducted in Portuguese.

### Instruments

We used semi-structured interviews that used the application of a timeline instrument to collect the data. The questions were asked in Portuguese and are included in Additional Material 1. The timeline instrument allows for a more global analysis of the individual’s life cycle, as the participants recount their history in chronological order. The timeline construction was divided into decades. The questions were based on the objectives of the general project. Furthermore, it can be used in conjunction with the interview [42]. A third party analysed the quality of the instrument. A pilot version was tested for viability and reliability before it was applied. After the test, a few small changes were made to the way the instrument was finally used.

### Data analysis

This study used the thematic analysis methodology developed by Braun and Clarke, which aims to identify, analyse and report themes or patterns within the data [43]. This approach allows for the construction of themes or analysis units in a broader way compared to other methods of analysis. To use this methodology, six steps are recommended: 1) familiarisation with the data; 2) assignment of preliminary codes; 3) organisation by themes; 4) review of themes; 5) definition and naming of themes; 6) production of the report. We used semantic analysis [43], a subtype of thematic analysis made from explicit meanings of the data, as this was the most appropriate method for this study.

All the interviews were audio-recorded and fully transcribed verbatim by members of the research staff. Theoretical saturation, the point at which no new information in relation to the themes is being observed, was defined by the researchers involved and was reached as of the 20th interview.

After being familiarized with the set of data through reading and rereading all the interviews, we proceeded to the coding in two phases [44]. The first consisted of the use of preliminary codes for the creation of provisional themes, divided according to the stages of the lifecycle, as defined legally in Brazil by the *Estatuto da Criança e do Adolescente* (Child and Adolescent Statute) [45], with childhood being 0–11 years of age, adolescence 12–18, and adulthood above 19 years of age on). In this phase, we used three types of coding out of the seven existing in book The Coding Manual for Qualitative Researchers [44]: 1) grammatical coding; 2) elementary coding; 3) exploratory coding. The codes that emerged identified the themes outlined in the study in respect of each life stage (childhood, adolescence, and adulthood).

The second phase was characterized by the deeper analysis and classification of the set of data, articulated with the methodological theoretical basis we had chosen, thematic analysis. Thereafter, we performed a more detailed coding using the software Nvivo 11 (QSR International, Melbourne, Australia, 2015) [46]. For this phase we used eclectic coding [44]. Each theme was created from sharing of 1/4 of the codes repeated among all the sources [44], that is, among all the interviews.

**Fig. 1:**
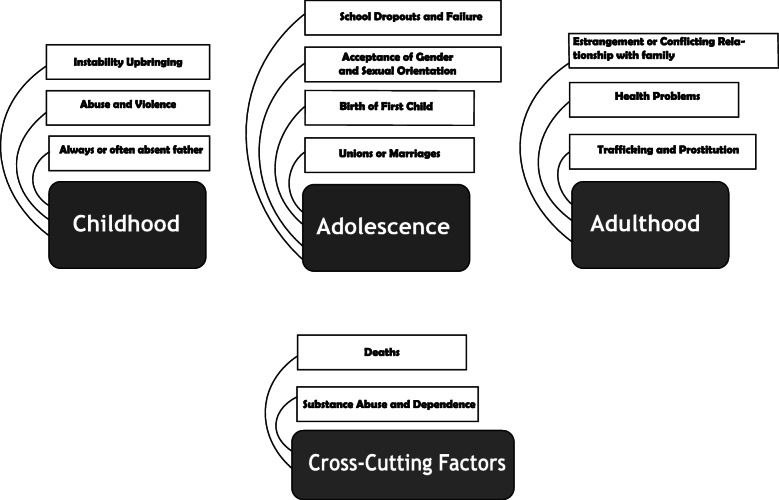
Theme Map

Due to its exploratory nature, the coding was inductive, i.e., the themes created are closely related to the datum itself [39, 43]. After following the six steps of the thematic analysis [43], we created the thematic maps (Fig. 1).

This study followed the checklists of the Consolidated Criteria for Reporting Qualitative Research (COREQ) [47] and Standards for Reporting Qualitative Research (SRQR) [48]. COREQ is a guideline with 32 items divided into three domains and is used in health care. Its objective is to improve the rigour and transparency of qualitative reporting, thereby promoting more credibility for the study [47]. The SRQR is a 21-item guideline that aims to improve the quality of qualitative research by using standard criteria [48].

## Results

Table 1 presents the characteristics of the participants. Twenty individuals between 21 and 62 years old participated in the study, of whom 13 were men (one transgender), and seven were women (two transgender). As regards family configuration, most of the participants were from large families (more than three siblings) and most of them had one child or more. More than half of the participants did not have a job (12), and among these individuals only one did not receive any aid from the government. Participants who reported working (8) had informal jobs, such as delivering fliers. Most of them, however, also reported receiving government aid. The majority of the respondents were single.

**Table 1:** Characterization of the participants

Respondents	Age	Sexual Orientation^a^	Work	Income Source	Children	Living with^b^	Marital Status	Number of Siblings
Respondent 1	20–30	Heterosexual	Yes	Government Aid	0	Street Friends	Single	12
Respondent 2	31–40	Homosexual	No	Government Aid	0	Partner	Cohabiting	3
Respondent 3	31–40	Homosexual	No	Government Aid	0	Street Friends	Engaged	1
Respondent 4	31–40	Heterosexual	Yes	Work	2	Alone	Single	2
Respondent 5	31–40	Heterosexual	Yes	Work	0	Alone	Widowed	10
Respondent 6	20–30	Heterosexual	No	Government Aid	0	Street Friends	Single	1
Respondent 7	20–30	Heterosexual	No	Government Aid	0	Alone	Single	5
Respondent 8	31–40	Homosexual	Yes	Work	3	Partner	Married	7
Respondent 9	41–50	Heterosexual	No	Government Aid	5	Alone	Single	1
Respondent 10	20–30	Heterosexual	Yes	Work	0	Alone	Single	6
Respondent 11	31–40	Heterosexual	No	Pension	3	Street Friends	Divorced	8
Respondent 12	51–60	Heterosexual	No	Government Aid	1	Street Friends	Separated	6
Respondent 13	31–40	Heterosexual	No	Government Aid	5	Shelter	Single	4
Respondent 14	31–40	Heterosexual	No	No	3	Alone	Single	5
Respondent 15	31–40	Heterosexual	Yes	Work	3	Alone	Single	3
Respondent 16	20–30	Heterosexual	No	Government Aid	1	Partner	Single	5
Respondent 17	20–30	Heterosexual	No	Government Aid	4	Partner	Cohabiting	0
Respondent 18	51–60	Heterosexual	No	Government Aid	3	Shelter	Single	15
Respondent 19	20–30	Heterosexual	Yes	Work	1	Shelter	Separated	11
Respondent 20	51–60	Heterosexual	Yes	Government Aid	2	Shelter	Separated	6

^a^ The definitions of gender, transsexuality and sexual orientation were based on the article by Joan Scott and a manual of theoretical concepts on this issue [49, 50]

^b^ The column “living in the street” describes whether the person lives with someone on the street or lives in a shelter, and is classified as alone, with street friends, or with a partner

Based on the analysis of the stages of the lifecycle, four themes emerged from the data relative to the histories and life trajectories of the participants. Figure 1 shows the theme map with its respective themes and subthemes subdivided by life cycle. The themes that stood out from the data were described and organized according to the stage of the life: 1- Childhood; 2 - Adolescence; 3 -Adulthood; 4 – Cross-cutting Factors.

### Theme 1: childhood

#### Subtheme 1.1: instability upbringing

About half of the participants reported a history of instability in their upbringing during childhood. They described recurring rearrangements of the family structure, with most of them reporting having been taken care of sometimes by their parents, sometimes by their grandparents and/or uncles and aunts, or even by other relatives or godparents. In some cases, these rearrangements were described as a very painful experience, often associated with a sense of rejection. In general, these instabilities were the result of the separation or death of the parents, mental disorders, or substance use by the caregivers.

“Because I don’t accept, until today I don’t accept [referring to her mother] that she didn’t raise me [...] left me at my grandmother’s to go to a dance one weekend and never came back to pick me up! [...] My grandmother raised me with a lot of love and care, but I don’t understand, I don’t get it, I have the view that where one can eat, two can eat; where one can starve, two can starve.” (Respondent 10)

#### Subtheme 1.2: abuse and violence

The respondents reported having suffered violence, mostly physical. The accounts highlight assaults within the family, many of which committed by a family member (usually the father) under the effect of some substance.

“Then, when I was little, my mother hit us a lot, you know? It was for us to rise in life, you know?^1^ [...] My way of thinking, you see? My family is kind of complicated. (Respondent 5)

#### Subtheme 1.3: always or often absent father

We observed in the reports about childhood that the respondents did not often mention experiencing life with their fathers. On the other hand, they highlighted the role of the female figure (mother or grandmother) in their upbringing. Many of them reported not having met their father or having had little contact with him.

“And my father, he wasn’t very present, he was in prison more often than not, he spent more of his life in prison than out of it, so he was not involved in with me growing up much.” (Respondent 10)

### Theme 2: adolescence

The respondents reported several difficulties regarding fragile school and work relationships during adolescence. Sexuality started too early, particularly for the girls. Family problems resulting from a compromised childhood also stood out. Within this theme, four subthemes emerged from the data.

#### Subtheme 2.1: school dropout and failure

More than half the respondents dropped out of school during adolescence, most during elementary school. There were also reports of failing and a history of participation in school equivalency courses. These events resulted mainly from factors associated with the use of psychoactive substances and low grades.

“Interviewer: And you studied up to which grade?

Respondent 16: Up to the eighth grade.

Interviewer: And what happened that meant you could not finish your studies?

Respondent 16: Crack!” (Respondent 16)

#### Subtheme 2.2: acceptance of gender and sexual orientation

Another factor that emerged from the data were social and family difficulties in respect of the acceptance of sexual orientation and gender, and the impact of this on the respondents’ acceptance of these things themselves. The respondents described prejudice from society and family before they reached their own acceptance.

“When I was 16, I admitted and accepted myself as homosexual, you know, I think it is particularly important! No, it was from 15 to 16, plus that I had psychological problems because of that, because I didn’t accept myself [...] (Respondent 3)

#### Subtheme 2.3: birth of first child

A considerable number of participants had their first child during adolescence. The main reports were from women who declared having first given birth in early adolescence. Some also had their other children in this period of life.

“I had my daughter when I was 15 [...]” (Respondent 8)

“My daughter was born when I was 17 to 18.” (Respondent 15)

#### Subtheme 2.4: unions and marriages

The union or marriage of very young intimate partners is a usual and accepted practice. This is more likely to be experienced by young women, and they account for the great majority of cases. For some, the union was forced on individuals because of pregnancy.

“After that I went back to Paraíba (a state in the Brazilian northwest) again, then, when I was 14, I got married!” (Respondent 8)

“[...] one week before I turned 18, they [family] found out I was pregnant and forced me to marry him.” (Respondent 4)

### Theme 3 - adulthood

Most respondents reported becoming homeless in adulthood. We observed difficulties in their family relationships, both regarding their family of origin and the family they raised. Two other sub-themes detected were health problems, and drug trafficking and prostitution.

#### Sub-theme 3.1: estrangement or conflicting relationship with family

One of the first factors to stand out in the data was in respect of family issues, particularly estrangement or conflicting relations with their family of origin, or sometimes both. As for the families they raised, one of the main subjects they talked about was loss of contact with their children, mainly because of separation from their spouses. Several participants stressed the psychological impact of this estrangement from their children, which triggered depression and, later, their moving to the street. The points stated in this sub-theme were the use of alcohol and other drugs, as well as sexual orientation, which the family did not accept, and fights.

“Me, my stepfather, we don’t talk for three years. In the house, [...] I am the last to eat, he fights a lot, humiliates a lot, he already humiliated me a lot, too.” (Respondent 6)

“[...] it’s about 3 years since I talked to them [the two sons], with my daughter I talk sometimes by Facebook.” (Respondent 8)

#### Subtheme 3.2: health problems

More than half of the respondents described physical and mental health problems. Some reported being HIV positive. There were also accounts of physical problems caused by accidents. The reports in respect of mental health problems were most commonly in respect of depression and substance use.

[referring to HIV] “When I was 17, I caught it, when I found out I was 18, 17 to 18! (Respondent 3)

“I was run over, lost my prosthesis, lost my glasses. [...] lost my teeth, then it was the domino effect, lost my job, lost the house, [...], lost the life that God gave me [...]” (Respondent 20)

#### Subtheme 3.3: trafficking and prostitution

Some respondents revealed having worked with drug trafficking and prostitution. To some, this started in adolescence. For most of them, however, this began in adulthood. Prostitution was more associated with the female and homosexual participants. Due to the need to survive plus a the lack of future prospects, a significant number of the participants used those precarious forms of work to generate some income.

“Then I knew that the only way to come to São Paulo, of course I had other means to come to São Paulo, but the easiest way to come was to prostitute myself, come to prostitute myself.” [...] As I wanted to come quickly, in the easiest way.” [...] Then I had to set my mind that I had to prostitute myself, when I was eighteen.” (Respondent 7)

“Trafficked, got it? Had a lot of women, I??? had money, I thought I had power, and I did. Only that then, I wasn’t noticing I was gradually destroying myself [...]” (Respondent 6)

### Theme 4: cross-cutting factors

Cross-cutting factors emerged from the data relating to these three stages of life. Two subthemes were also observed in the themes mentioned above. Some respondents reported having experienced situations related to death and substance use in childhood or early adolescence.

#### Subtheme 4.1: deaths

The reports highlight grief for the deaths of people close to the respondents (mainly parents and grandparents) with whom they had a bond of dependence (relationship or income). This subtheme presented high intensity, as it often elicited a sequence of significant changes in the life of the participant.

“Well, my family life from 0 to 10 years of age, I lost my father when I was 4, my mother when I was 6, my grandmother when I was 8, it was a sequence.” (Respondent 3)

“It was soon, when my mother passed away, [around the age of 40], because my mother, I was everything, you know, I earned, I didn’t earn, but it was everything, [...] and then the guys, when my mother passed away, threw me out of the house, my brothers, you know, then my father left with me at the time [...]” (Respondent 20)

#### Subtheme 4.2: substance abuse or dependence

There were reports of relatives with a history of substance abuse, with alcohol and crack use standing out. We observed a history of several admissions in many cases to therapeutic communities.

“At 19 years old I started to use cocaine, and three years ago I started to use crack [..] It was then that I really started living on the street...”. (Respondent 14)

“Why I lost my job? Because I didn’t smoke during the week, I started to smoke, I started to miss work, [...] then it gets to a point when it’s enough, you know, then I was fired.” (Respondent 10)

## Discussion

This study explored the characteristics of the interpersonal relations of homeless people who use alcohol and other drugs in three stages of human development: childhood, adolescence, and adulthood. The contents that emerged from the data corresponding to the respondents’ childhood are closely related to their birth family. Regarding adolescence, with the exception of the subtheme *school dropout*, the others were related to sexuality. In Adulthood, family issues return as the main themes, most often related to conflict, or the loss of contact with family members which, according to the subthemes that emerged, was not well established in childhood. We also identified cross-cutting themes that permeated the different stages in the life of the participant. The interpersonal relationships of the participants are connected to several social problems that stem from social exclusion in the political, social, cultural, and economic domains.

Bronfenbrenner’s ecological systems theory argues that human development is a phenomenon of continuities and changes deeply influenced by the environment [51]. Changes in environment have great potential to change the direction of an individual’s development Such transitions might be normative, those naturally expected (puberty, starting school, new job), or non-normative, characterized by unexpected events (deaths, divorces) [51, 52]. In our study, the normative ecological transitions took place earlier than expected (mainly in adolescence) and the non-normative ones contributed to a development filled with problems. From childhood to adulthood, the ecological transitions promoted significant changes at the level of the microsystem (the individual’s immediate environment comprising family, peers, school, neighbourhood, church etc.) as a result of the environment of the interviewees being disorganized.

### Childhood

The respondent’s family structure was affected by a series of rearrangements and the frequent absence of the father, in addition to violence and abuse from their fathers or relatives. As Bronfenbrenner emphasizes, family is the main context in which human development takes place [53]. In this respect, the relationships established, developed, and destroyed throughout life are profoundly shaped by this period.

We should point out the interconnection between social exclusion and development throughout life, mostly in respect of the social dimension. In this regard, a study of children exposed to experiences of social disadvantage, such as family instability and abuse, demonstrated that they were more prone to display behaviours such as stealing, and selling or using drugs in adulthood than those who were not exposed to social these experiences [37]. Social disadvantage in the family might have a number of different consequences, such as the loss of jobs, that can cause high levels of parental stress, in turn negatively affecting the daily care of the child, and promoting a domino effect [54, 55].

In respect of the absence of the father, our results draw attention to a serious problem in Brazil. A report from the *Conselho Nacional de Justiça* (National Council of Justice) [56], based on a 2011 census by the *Instituto Nacional de Estudos e Pesquisas Educacionais Anisio Teixeira* (INEP) – (a research agency linked to the Ministry of Education) revealed that over 5 million children do not have their father’s name on their birth certificate. The negative consequences of paternal absence may include deficits in cognitive development associated with behavioural disorders, chiefly concerning the development of self-esteem and creating healthy emotional bonds [57, 58].

### Adolescence

The subthemes we detected in adolescence were school dropout and failure; acceptance of gender and sexual orientation; birth of first child; unions or marriages. A cross-sectional study with adolescents in the USA demonstrated that school problems during adolescence are predictive of homelessness in early adulthood [59]. Another study with adolescents between 12 and 14 years old [60] revealed that episodes of running away from home to live on the street might have a significant impact on school performance in the long term. In this regard, we highlight the result of a Canadian study [61], in which a history of homelessness was associated with school dropout. This theme is extremely important, as low educational levels might hinder the social mobility of individuals [62], and affect a whole generation [63]. Marriage and pregnancy in adolescence are considered a public health issue [64] and are associated with, predominantly for the woman, mental health and educational problems, in addition to obstetric risks [65, 66]. In Brazil, the risk of pregnancy is 16 times higher among adolescents who live in *favelas* than among those from middle class families [67]. These data stress the deep, constant social inequality that many developing countries face.

A 2108 study by Garcia demonstrated that conflicts with the birth family related to the breach of heteronormativity were a determining factor for those individuals in respect of living on the street and becoming homeless, although this does not happen in adolescence [68]. If we conceive family as a fundamental institution for life in society, the LGBTQI+ population might be ostracized by being expelled from that institution, a process that might in turn lead to the weakening of interpersonal relationships.

### Adulthood

Health problems, drug trafficking and prostitution were the main subthemes that emerged. We observed a strong connection with the previous phases, since the participants reported several experiences related to social exclusion across the four dimensions.

Studies emphasize family conflict as one of the most relevant factors in respect of individuals becoming homeless [32, 69]. The study by Mabhala et al. [37] corroborates the themes raised in this study. The authors describe the process of becoming homeless, in which there is first a decrease in resilience, developed through the adversities associated with abusive environments, and characterized by abuse and violence in childhood. Second, is the involvement in non-adaptive behaviours (mainly in terms of substance use) and problems with authorities. Finally, the individual leaves home to live on the streets, along with the total collapse of family relationships [37]. Varanda and Adorno share this view. People who live in poverty experience fragile social bonds which can strengthen or break, depending on their experiences over their life course [32].

As regards health, research demonstrates higher mortality rates among HP [17, 70], in addition to higher rates of infectious diseases, substance use and mental health disorders. Therefore, we should consider not only how precarious the street can make a person’s health, especially if it was already fragile before going to the street, but also how much the fight for survival and dignified life conditions influence their staying on the streets.

A study among women who experienced homelessness indicated that they used prostitution as a way to survive, and ended up trapped in a vicious circle that kept them on the street, with drug dependence playing a significant role in maintaining this circle [71]. In our study, prostitution and drug trafficking are viewed as work modalities closely associated with the economic and political dimensions of social exclusion [31], being an attempt to survive out of the social norm. As a result, this situation increases the stigmatisation of these individuals, being branded as homeless, drug users, drug dealers and prostitutes.

### Cross-cutting factors

The impact of the death of people close to the respondents on their human development stands out in their statements. These events were often related to a significant change in the life of the individuals, favouring homelessness. A study among HP found that the loss of family members and relatives at an early age contributed to the individual becoming homeless [72]. Another study highlighted the death of parents as a precipitator of housing instability among young American women from a region of extreme poverty [73].

The use of drugs in this study is associated with the frailty of family and occupational bonds prior to the street situation [74, 75]. Dependence might be the result of a process that crosses the whole life course and has the potential to favour homelessness at any point of the cycle. To some, homelessness seems to intensify abuse and the consequences of dependence, while for others it appears to be the trigger for homelessness or its maintenance. However, for most of the participants in this study, both phenomena happen in a circular feedback manner. Homelessness and dependence increase the likelihood of social exclusion, and result in health difficulties and reduced wellbeing [28].

The whole set of data in our study emphasize several life conditions related to successive estrangements that can be cumulative over time, resulting in problematic effects for the developing individual.

### Limitations and strengths of the study

Given that the study is retrospective, and the participants are adult, the lack of precision and details of childhood and adolescent events varies according to the age of the respondent and their memory. However, such variability favours the reporting of events that have particular significance for the interviewee. In respect of another aspect of the study methodology, we should bear in mind that the answers of the participants were similar, independently of the time and venue of the interviews, and hence did not influence the results. Considering the self-report nature of the study, factors that individuals found it difficult to talk about or were unwilling to talk about, such as poverty and ethnicity issues, may not have been given enough prominence. In respect of strengths of the study, we highlight the originality of the timeline perspective of this study, as it provides guidelines for understanding the factors that precede the phenomenon of homelessness. Another strength regards the key informants, as some respondents demonstrated a strong bond with these individuals and great respect for their work, which resulted in the respondents being more willing to be open and honest in respect of the information they provided in the interviews.

## Conclusion

The interpersonal relations of the participants in this study are linked to the four dimensions of social exclusion (economic, cultural, political, and social). The results suggest that these relations were impaired by successive break-downs or the ending of significant relationships which had serious and problematic effects on the development of the individual, starting in adolescence and continuing in adulthood. Childhood was characterised by family instability and violence. In adolescence, problems seem to have intensified alongside the inherent challenges of this period of life and, added to the new challenges of adulthood, led to the individual ending up homeless. Substance abuse and dependence, as well as the death of close people, were factors that promoted social exclusion in all periods of life, and potentialised pre-existing or concurrent vulnerabilities. Policies and interventions should consider the multiplicity of vulnerabilities that accumulate across the life course of a homeless individual in order to address their condition from a broader perspective. Knowing the nature of the disruption of interpersonal relationships at each stage of life will help decision makers to develop public policies that favour the strengthening of these people’s bonds and help to avoid homelessness. Children and adolescents with a history of disruptions similar to those described in this study should receive special attention from caregivers and authorities due to their increased vulnerability, with interventions to strengthen bonds and prevent them ending up on the street. In respect of adulthood, interventions must be at the governmental level and focus on interdisciplinary health care to try to reduce the impact of earlier family instability; provide greater social assistance and psychological help to improve interpersonal relationships; and work to provide financial independence for the homeless so that they can secure housing.

## Supplementary Information

**Additional file 1.** Guideline to the Construction of Timeline.

## Data Availability

All materials are available from the corresponding author.

## Abbreviations

HP: Homeless people
OECD: Organization for Economic Co-operation and Development
WHO: World Health Organization
COREQ: Consolidated Criteria for Reporting Qualitative Research
SRQR: Standards for Reporting Qualitative Research
INEP: Instituto Nacional de Estudos e Pesquisas Educacionais Anisio Teixeira - A research agency linked to the Ministry of Education
LGBTQ+: Lesbian, gay, bisexual, transgender, and queer; the “plus” intended to be a comprehensive representation of sexual orientations and gender identities

## Footnotes

1 It’s a Brazilian expression like “We were supposed to rise in life, right”. Many people believe in the false idea that educating is synonymous with hitting.
