# Supplementary material for: Interpersonal relationships and drug use over time among homeless people: a qualitative study
Source: BMC Public Health. 2020 Nov 19;20:1746. doi: 10.1186/s12889-020-09880-2 (PMC7678275; doi:10.1186/s12889-020-09880-2)
Supplement: Supplementary file 1 — Additional file 1. Guideline to the Construction of Timeline. [file 12889_2020_9880_MOESM1_ESM.docx]

**ADDITIONAL FILE 1**

**Guideline to the Construction of Timeline**

First the interviewer collected the sociodemographic data (Table 1).

The timeline construction was divided into decades. The questions were based on the objectives of the general project. Additional questions were asked when the respondent was not interacting or was embarrassed to provide some information about their past.

**Set 1 - Sociodemographic Data**

Age:

Do you follow any religion?

Do you have any disability? (physical, psychiatric or both?)

Do you work? Do you have any income source?

What’s your city of birth?

How do you identify your gender?

How many siblings do you have? Are you the oldest or youngest?

Do you have children?

Marital status?

Do you identify with any race or ethnicity?

Who do you live with? (Family on the street, alone on the street, friends on the street, in a shelter).

***Life Cycle Questions:***

**Childhood**

What were your family relations like from ages 0 to 10?

What were your school relations like from ages 0 to 10?

What were your friendships like from ages 0 to 10?

**Additional questions**

What’s the history of your first name?

Are your parents alive?

Did you have friends in your childhood?

What was the relationship with your parents like?

Were you a child who played?

Did you move to a new house very often?

What good and bad things do you remember most about your childhood?

**Adolescence**

What were your family relations like from ages 10 to 20?

What were your relations with work like from ages 10 to 20?

What were your personal relationships like from ages 10 to 20? Sweethearts, etc.?

What was your behavior like regarding the use of alcohol and other drugs like from ages 10 to 20?

**Additional questions.**

Did you continue studying? Was the school public or private?

Do you have children: do they have the same father?

How old were you when you started working?

Did your parents have problems with alcohol and/or other drugs?

**Adulthood**

What were your family relations like from ages 20 to 30?

What were your school relations like from ages 20 to 30?

What were your relations with work like from ages 20 to 30?

What were your affective relations like from ages 20 to 30? Sweethearts, etc.?

What was your behaviour like regarding the use of alcohol and other drugs from ages 20 to 30?

**Questions unrelated to the Life Cycle**

When did you become homeless?

What’s your relationship with the present services? (Health, Assistance, NGOs, Churches, etc.)

What’s your routine on the street?

How do you see yourself in the future? Five or ten years from now?

Tell me something you do very well, something you are good at. A talent.

What are your dreams?
